# Exploring the stakeholder‐informed adaptation of a goal‐based psychosocial intervention for people with dementia and their caregivers in diverse services and communities in the UK

**DOI:** 10.1002/alz70858_096547

**Published:** 2025-12-24

**Authors:** Jessica Budgett

**Affiliations:** ^1^ Queen Mary University of London, London, London, United Kingdom

## Abstract

**Background:**

Dementia interventions are often adapted for various contexts, cultures and languages. A critical emerging area of research in dissemination and implementation science is understanding what adaptations are made, why and when they occur, and their impacts. NIDUS‐Family is a clinically and cost effective manualised psychosocial intervention tailored to the personal goals of people living with dementia and their families, aiming to support living well at home for longer. This study aimed to explore and characterise the adaptations required to implement NIDUS‐Family in UK primary, secondary and third sector services. To address inequalities in post‐diagnostic support, we also aimed to conduct a linguistic and cultural adaptation to our local Bengali community in East London.

**Method:**

The core elements of NIDUS‐Family deemed essential to effectiveness were identified and defined by our implementation group of academics, clinicians and PPI members, building on trial and process evaluation evidence. We conducted a series of focus groups with PPI members, service staff and other key‐stakeholders within five primary care practices, two secondary care memory services, and the Alzheimer's Society. Semi‐structured topic guides were co‐produced and derived from the Consolidated Framework for Implementation Research (CFIR), with a focus on the sites’ inner and outer contexts that enable or constrain implementation and modifications to the intervention's peripheral elements to enhance contextual fit. An additional focus group and individual interviews engaged family carers and people with dementia from the Bengali community and staff members in their local memory service. Focus group and interview transcripts were thematically analysed. All adaptations made during the study will be recorded using the Framework for Reporting Adaptations and Modifications (FRAME).

**Result:**

Findings from the thematic analysis will be presented, as well as our methods of using evidence to develop a tailored adaptation plan for each service and Context‐Mechanism‐Outcome (CMO) configurations to iteratively develop our programme theory.

**Conclusion:**

Systematically characterising and reporting adaptations made to dementia interventions is crucial to enhancing their contextual fit. By engaging diverse stakeholders, we can ensure interventions are both culturally and contextually relevant. These findings will inform the wider scale implementation of NIDUS‐Family.

